# Effects of the Communities that Care (CTC) Prevention System on Youth Violence Outcomes in Two Violence-Impacted Denver Communities

**DOI:** 10.1007/s12103-025-09811-0

**Published:** 2025-07-12

**Authors:** Beverly E. Kingston, Sabrina Arredondo Mattson, Jani S. Little, Christine M. Steeger, Eric J. Sigel, Uriel Lomelí Carrillo, Dave Bechhoefer, Susanne Argamaso, Ashley D’Inverno

**Affiliations:** 1 Institute of Behavioral Science, University of Colorado Boulder, 1440 15th St, Boulder, CO 80309 USA; 2 Adolescent Medicine, Children’s Hospital Colorado, University of Colorado School of Medicine, Aurora, CO USA; 3 Division of Violence Prevention, National Center for Injury Prevention Control, Centers for Disease Control and Prevention, 4770 Buford Hwy, NE, MS S106-10, Atlanta, GA 30341 USA

**Keywords:** Youth violence, Communities that care, Prevention strategies, Interrupted time series, Difference in differences

## Abstract

Preventing and reducing youth violence remains a significant public health challenge for urban marginalized communities across the country. This project used a Communities That Care (CTC) approach to build a community-level prevention infrastructure across two Denver, Colorado communities experiencing high-burden, with a primary aim of reducing youth violence. To examine the impact of the CTC system on youth violence outcomes, we assessed within- and between-community effects using two quasi-experimental approaches—Interrupted Time Series (ITS) and Difference in Differences (DiD) analyses with synthetic controls. The primary violence outcome was official youth (aged 10–24) annual arrests for violent offenses, measured for five years prior to the intervention (2012–2016) and for five years after the start of the intervention (2017–2021). ITS results show that there was an intervention effect in Community B as demonstrated by lower arrest rates early in the intervention period, suggesting that the mobilization period was impacting the intervention community with respect to the arrest rates. There were no significant effects in Community A. The DiD analysis with a synthetic control confirmed that the arrest rates in Community B were significantly lower than in the synthetic control community during the intervention years. The CTC approach and the community-level prevention system implemented in intervention Community B was associated with a reduction in youth arrests for violent offenses during the intervention period; no intervention effects were detected in Community A. Additional research is needed to understand why the intervention was effective in Community B, but not Community A.

**Supplementary Information:**

The online version contains supplementary material available at 10.1007/s12103-025-09811-0.

## Background

 Preventing and reducing the risk for youth violence remains a significant challenge for communities across the country. Youth violence (e.g., homicides, fights with injuries, aggravated assaults, firearm violence) is a major cause of mortality and morbidity among youth, particularly among urban marginalized communities (Martin et al., 2022; Nation et al., 2021; Sheats et al., 2018 ). The Centers for Disease Control and Prevention (CDC) reports that homicide is the third leading cause of death for young people between the ages of 10 and 24, the leading cause of death for Black youth, the second for Hispanic youth, and the third for White and American Indian and Alaska Native youth (David-Ferdon et al., 2021; CDC, 2023 ). Recent studies show a 30% increase in homicides during the first year of the COVID-19 pandemic in 2020, compared to the year prior, and continued increasing homicide rates into 2021 across urban cities (Jossie et al., 2022; Rosenfeld & Lopez, 2020, 2022 ). Similarly, there is evidence of increased gun violence and firearm homicides during the pandemic (Kegler et al., 2022; Kim & Phillips, 2021; Simon, Kegler, et al., 2022; Ssentongo et al., 2021 ). These higher homicide and gun violence rates are hypothesized to be at least partially attributed to a surge in firearm purchases, police violence protests, and other contextual factors early in the pandemic such as increased economic and psychological stressors, as well as alcohol and substance use (Rosenfeld & Lopez, 2020; Ssentongo et al., 2021; West et al., 2023 ). The economic burden of youth violence is substantial, costing an estimated $122 billion annually (in 2020) in direct and indirect costs of homicides, nonfatal physical assault-related injuries, and negative consequences for families, schools, and communities (Peterson et al., 2023 ). Given the significant public health impact of violence on young people’s development and community functioning, effective ways of preventing and reducing youth violence are clearly needed. 

### Youth Violence in Communities Experiencing High-Burden and Opportunities for Prevention

The high prevalence and many negative consequences of violence are magnified for people living in communities characterized by social and economic disadvantage. These communities are less likely to have the opportunities, resources, and supports that promote healthy development, and they are more likely to expose youth to additional risk factors, such as violence, community and family disorganization, as well as readily available drugs and alcohol (Elliott et al., 2006; Gershoff & Aber, 2006; Kingston, 2011). Further, people from racial and ethnic minority groups tend to experience conditions of concentrated disadvantage (Friedson & Sharkey, 2015) along with other negative behavior and health consequences. Results from the 2021 Youth Risk Behavior Survey (YRBS) showed that most youth from racial and ethnic minority groups (e.g., American Indian or Alaska Native, Black, and Hispanic) were more likely to witness community violence and to report gun carrying compared to their White peers (Harper et al., 2023; Simon, Clayton et al., 2022). In turn, the differential exposure to violence by race and ethnicity might increase disparities in other types of violence-related morbidity and mortality because of heightened stress and adversity (Armstead et al., 2021). In particular, compared to White youth, Black youth are often exposed to higher rates of violence (Martin et al., 2022). Additionally, Black adolescents and those from other racial and ethnic minority groups may also have elevated rates of internalizing and externalizing symptoms of psychological distress (Gaylord-Harden et al., 2011; McGee, 2003), substance use and poor grades (Bradshaw et al., 2013), and greater reported hopelessness and a sense of shortened mortality (Burnside & Gaylord-Harden, 2019). Violence also negatively affects communities by reducing opportunities for positive neighborhood and school engagement and social relationships (Cheung, 2023; Lin et al., 2020). The many negative consequences of youth violence motivate the objective of this study, which was to implement prevention programs in two urban communities experiencing high burdens of violence, with the goal of significantly reducing youth violence, thereby improving youth behavioral health and well-being.

### A Public Health Approach for Reducing Youth Violence in Communities Experiencing High-Burdens of Violence

A large body of research supports using a coordinated, comprehensive public health approach to reduce risk factors and to enhance protective factors at the individual, family and peer relationship, school, community, and societal levels of the social ecology (U.S. Department of Health & Human Services, 2001; Jenson & Fraser, 2011; Kingston et al., 2021; Ridgeway, 2014). A public health approach to reduce youth violence: (1) is developmental (birth to young adulthood), (2) addresses varying levels of risk (universal, selective, indicated preventive interventions), and (3) aligns evidence-based preventive interventions across multiple social contexts (individual, relationship, school, and community) (Kingston et al., 2016a, b; McCrea et al., 2019; Sullivan et al., 2015).

Youth violence prevention strategies that focus on the community level, or outer layer of the social ecology (Kingston et al., 2021), may be key to addressing the disproportionate burden of violence long experienced by communities at increased risk for youth violence (Zimmerman & Messner, 2013). Community-level prevention focuses on the social, economic, and environmental characteristics of settings (e.g., schools, workplaces, and neighborhoods) that may be risk or protective factors for youth and community violence. Though less is known about the effectiveness of interventions targeting community-level factors (David-Ferdon & Simon, 2014; Fagan & Catalano, 2013), these interventions are likely an essential complement to individual- and relationship-level strategies, have an impact on a large number of people, and are necessary for a comprehensive prevention approach. However, the prevention science field lacks a sufficient evidence base of effective community-level youth violence prevention approaches (David-Ferdon et al., 2016).

The current project uses the Communities That Care (CTC) approach to build a local prevention infrastructure (i.e., a coordinated system of research, innovation, and service delivery), which engages community members to mobilize and use a science-based approach to prevent violence and improve youth outcomes (Fagan & Hawkins, 2012; Hawkins et al., 2008). Although CTC has been tested and implemented in several community settings, most of the research shows its success in smaller, primarily White communities (Brown et al., 2009; Feinberg et al., 2007, 2010). CTC has recently been tested in more diverse international areas (e.g., Australia and Germany; Röding et al., 2021; Rowland et al., 2022; Toumbourou et al., 2019) and urban communities that may experience challenges of social and structural inequities (Brady et al., 2018). This project contributes to the CTC research base and the broader context of effective community-level prevention strategies aimed at reducing youth violence by describing the modifications and lessons learned in the implementation of CTC in urban U.S. communities. Further, this study includes CTC implementation during the first year of the COVID-19 pandemic. To our knowledge, few manuscripts report on implementation of community-level prevention strategies and youth and community violence trends during the pandemic; thus, this study also adds an important context to the youth and community violence and prevention literature base.

#### CTC Theoretical Rationale

This study implemented and evaluated a modified version (described below) of CTC in two Denver, Colorado communities experiencing high-burden. The CTC five-phase process was used as the guiding framework to engage and organize these two communities to develop a prevention infrastructure and build evidence-based supports that focused on community- and policy-level prevention strategies (Fagan & Hawkins, 2012; Hawkins et al., 2008). CTC includes the following five-phase process: (1) *Get Started* – assessing community readiness to undertake collaborative prevention efforts; (2) *Get Organized* – getting a commitment to the CTC process from community leaders and forming a diverse and representative prevention coalition; (3) *Develop a Profile* – using epidemiologic data to assess prevention needs; (4) *Create a Community Action Plan* – choosing tested and effective prevention policies, practices, and programs based on assessment data; and (5) *Implement and Evaluate*. Each phase has specific milestones and benchmarks and includes training and technical assistance by a certified CTC trainer (Quinby et al., 2008).

In traditional CTC, the community coalitions can select and implement evidence-based programs that target their prioritized risk and protective factor needs (e.g., including individual and relationship domains). This project modified CTC to ensure that the Community Action Plan developed in Phase 4 included only the implementation of community- and policy-level prevention strategies designed to prevent youth violence. While there is an acknowledged gap in the field of evidence-based approaches to youth violence prevention at the community and societal levels, these strategies have a sound theoretical basis and preliminary empirical support (Puddy & Wilkins, 2011). With sufficient reach and dosage, the strategies can have a community-level impact by reducing serious forms of youth violence. Additionally, it is expected that by implementing these strategies within the supportive infrastructure of CTC, they are more likely to fit the local context and be well implemented.

### Study Purpose

The purpose of this study was to (1) determine within-community violence trends before and after the intervention, (2) test the effects of CTC strategies in the intervention communities compared to synthetic control groups built from the remaining Denver communities that did not experience the intervention, and (3) describe the implementation process evaluation in the two intervention communities (i.e., readiness capacity, coalition functioning, and implementation reach). Results of this study contribute to the CTC and broader community-level violence prevention research base by documenting its effectiveness in reducing youth violence as well as by describing the modifications and lessons learned through the implementation of CTC in violence-impacted urban communities.

## Methods

### Overview of Study Design

This study aimed to reduce youth violence in two urban Denver communities experiencing high-burden that had different levels of readiness to implement community-level prevention efforts. The project implemented and evaluated the CTC community-level prevention system that delivers community- and policy-level violence prevention strategies matched to local needs. The project used a CTC approach to build a local prevention infrastructure, which included a coordinated system of research, innovation, training, and service delivery at the community level. This infrastructure included forming a coalition of residents and stakeholders, collecting and analyzing data to prioritize risk and protective factors, creating an action plan, monitoring implementation and evaluating its impact. This study used two quasi-experimental approaches, Interrupted Time Series (ITS) and Difference in Differences (DiD), to evaluate changes in youth arrests for violent offenses within two intervention communities. Outcomes were examined within each intervention community for five years prior to the intervention (2012–2016), and for five years after the start of the intervention (2017–2021). The study was reviewed and approved by the Institutional Review Board (IRB) at the University of Colorado Boulder.

### Community Sample Selection

Two communities in Denver, Colorado composed mostly of people from racial and ethnic minority groups were selected as the two study intervention sites because of their high concentrations of violent crime and economic disadvantage. In addition, there were practical considerations, including prior familiarity and good working relationships between the research team and community leaders. The two communities will be subsequently referred to as Community A and Community B to preserve confidentiality. Youth arrest rates significantly exceeded the national average youth arrests for violence offenses per 100,000 youth (City and County of Denver, Denver Police Department/Data Analysis Unit). From the U.S. Census Bureau’s American Community Survey (ACS), 5-year estimates for 2012–2016, in Community A, 11% of families were below the poverty line, 12% of the population was over 65 years of age, 23% identified as Black or African American only and 58% identified as Hispanic. In Community B, 19% of families were below the poverty line, 15% of persons were over 65 years of age, 39% identified as Black or African American only, and 27% identified as Hispanic.

Both selected communities, A and B, had been participants in the CDC-funded Youth Violence Prevention Center-Denver (YVPC-D) since 2011. At that time, the site selection criteria for YVPC-D included high rates of youth violence and risk factors for violence, as well as an active neighborhood organization which could serve as the backbone for the prevention infrastructure created through CTC. Both Communities A and B were recommended by Denver’s Crime Prevention and Control Commission as neighborhoods of concern for elevated youth violence and gang involvement. Likewise, both had established neighborhood groups that could serve as the infrastructure for CTC. As such, Community A had been implementing CTC and a package of evidence-based programs between 2011 and 2016 while Community B served as its comparison site during that time. Implementation of CTC prior to the present study may account for the lower and stable arrest rates in Community A three years preceding the current intervention. Established relationships with these two communities eased the 2016 transition into community- and policy-level work.

### Description of the CTC Prevention System and the Intervention Community-Selected Strategies

CTC provided the backbone infrastructure for the two communities that implemented the community-level prevention strategies. Intervention Community A created its coalition and had been using CTC as its driving support and oversight mechanism for implementation of evidence-based programs and strategies in the prior CDC-funded initiative (2011–2016). Community B formed its coalition in 2016, and they elected to use a shortened version of the traditional in-person training model.

As previously described, this project used modifications to CTC Phase 4 where Community Action Plans were established. Rather than communities selecting from a wider range of evidence-based programs and strategies across the spectrum of domain areas (including individual and family), the communities were presented with a menu of theoretically-based violence prevention strategies designed to have reach and dosage specifically at the community level of impact. Each CTC coalition used their neighborhood data results (collected in 2015 as part of the prior CDC-funded project) to prioritize needs and determine which strategies to implement. Table 1 lists the strategies selected based on the risk and protective factors prioritized by each neighborhood.

**Table 1:** Youth and community violence risk & protective factors and community-selected strategies to address identified factors

	Community A	Community B
Risk Factors	• Low neighborhood attachment/ Community disorganization • Family management problems • Lack of commitment to school/ truancy	• Low neighborhood attachment/ Community disorganization • Family management problems • Early & persistent problem behavior/ Friends engaging in antisocial behavior
Protective Factors	• Recognition for prosocial involvement • Knowledge of community resources	• Recognition for prosocial involvement • Opportunities for prosocial involvement
Community Selected Strategies	• “Power of One” media campaign • Mini grants initiative • Positive Recognition Campaign	• “Power of One” media campaign • Promoting Alternative Thinking Strategies (PATHS)/Community SEL
CTC/Included Strategies	• Social Development Strategy • Violence, Injury Protection and Risk Screen (VIPRS)	• Social Development Strategy • Violence, Injury Protection and Risk Screen (VIPRS)

Note: SEL = social emotional learning

### Community-Level Violence Prevention Strategies

As described above, the five community-level violence prevention strategies implemented in this study were selected based on prioritized risk and protective factors in each community. Each strategy was developed using a theoretical rationale and was based on existing empirical evidence, fit to the community, and motivation of the community to implement the strategy (Kingston et al., 2021; Puddy & Wilkins, 2011).

#### Strategies Implemented in both Communities

The *Power of One (PO1) Media Campaign* is a youth-led media campaign that used a mix of digital video advertising, public events, and social media content with messaging to engage youth in violence prevention efforts and increase youth attachment to their neighborhoods (CDC, 2020; Linkenbach, 2003). This strategy was developed to address the risk factor *low neighborhood attachment* in both intervention communities.

The *Social Development Strategy* (SDS) is a research-based youth engagement and positive youth development framework that is part of CTC (Haggerty & McCowan, 2018). When youth are provided opportunities to engage, increase their skills, and then recognized for their efforts, they will bond to the socializing agent (e.g., person, group). For youth, this bond can encourage the desire to listen to and live by the socializing agent’s standards. If youth bond to individuals with healthy, socially responsible beliefs and behaviors, the bond can positively influence behavior creating a protection against risk for violence. After participating in a SDS training, the coalition embedded the SDS into the community level prevention strategies (e.g., organizations applying for mini grants were required to demonstrate how they provided the opportunities, skills and recognition aspects of the SDS in their programming for youth).

Finally, the *Violence*,* Injury Protection and Risk Screen* (VIPRS) is a risk screening tool designed for use in primary health care settings. This tool creates a standard and feasible way for pediatricians to identify youth who are at risk for future serious violence perpetration in the year after screening positive during a primary care visit (Sigel et al., 2011; Sigel & Harpin, 2013). Youth who are identified as posing high or medium risk for future violence perpetration are then referred to services to address their needs.

#### Strategies Implemented in Community A

 The *Community Mini-Grant* strategy provided Community A organizations and initiatives small, one-year financial stipends (grants) to increase community engagement in the neighborhood, and to increase connectedness among community members to the amenities, businesses, community organizations, and services available to them. The funded programs were required to directly involve youth with the intention of helping them to become more involved in neighborhood activities. All grant proposals were reviewed by panels of community members, including community youth, and stipends of up to $2,500 were awarded. From 2018 to 2021, there were four rounds of grants awarded for a total of 47 awards. The average grant amount across all four years was $2,095. This strategy was developed to target the *low neighborhood attachment* risk factor that was prioritized by Community A’s coalition.

Next, the *Positive Recognition Campaign* was an effort to highlight and positively recognize youth ages 10–21 who have made meaningful contributions and connections to the community in Community A. The campaign was originally developed and implemented as an individual-level strategy in the previous CDC grant to address the *low neighborhood attachment* risk factor and was revived as a community-level strategy after the Community A coalition expressed interest in continuing to support neighborhood youth through public acknowledgement of their accomplishments. The goal of this strategy was to strengthen prosocial bonds and to build pride among youth for their community (Catalano & Hawkins, 1996).

#### Strategies Implemented in Community B

The *Promoting Alternative Thinking Strategies* (PATHS)/Community Social Emotional Learning (SEL) is a universal, SEL program for elementary schools designed to improve student behavior, reduce classroom disruptions, and increase academic engagement and achievement (Greenberg et al., 1998; Malti et al., 2011). The program was selected by the Community B coalition to address the prioritized *early and persistent problem behavior* risk factor. The coalition wanted to broaden the reach of PATHS by developing a community wide SEL approach that includes a common SEL language and provides resident youth with multiple opportunities to have these competencies reinforced through interactions with adults and peers in different community settings. In addition to three neighborhood elementary schools, facilitators in after-school and summer programs, and community sports programs were trained to provide SEL-themed sessions to youth ages 6–18.

### Implementation Process Evaluation

Community strategy implementation was monitored closely with standard process evaluation techniques. The process evaluation included (a) a readiness and capacity assessment of the CTC community board and key community partners to build prevention infrastructures, and (b) fidelity monitoring of CTC and the implementation of the selected community-level prevention strategies to determine how program activities were delivered and mechanisms through which CTC and the selected community-level prevention strategies functioned.

To assess the readiness and capacity of local coalition functioning to build prevention infrastructures, three types of data were collected in each community: coalition meeting evaluations, annual community coalition surveys, and a CTC milestones and benchmarks tool.

### Youth Violence Outcomes

Annual youth arrest rates for violent offenses were used to evaluate the effectiveness of the strategies implemented within each intervention community. Arrest incident data were provided by the Denver Police Department, and they spanned the five years prior to the intervention (2012–2016) and the five years after the start of the intervention (2017–2021). The data included arrests of youth ages 10–24 for murder and non-negligent manslaughter, robbery, aggravated assault, and rape that occurred within each of the 76 communities in Denver. Arrests for these offenses were used to create an additive violent crime index, which is simply the total number of arrests within these categories by year by community (Osgood, 2000; Yu et al., 2020).

To create annual youth arrest rates for each community, the numbers of annual arrests needed to be divided by the estimates of the annual youth populations, which were gathered from the ACS five-year tract-level data disseminated by the National Historical Geographic Information System (NHGIS) (Manson et al., 2022). The tract-level estimates were summed to form the community-level youth population estimates for each community for each year. However, to reduce year-to-year random fluctuations that might overly influence arrest rates, the ten-year time series of population estimates for each community were smoothed with a quadratic function before they were used as the denominators of the arrest rates. Thus, the annual arrest rates were calculated as the numbers of youth arrests for violent offenses reported in a community during a calendar year divided by the smoothed annual youth population estimates, and finally the rates were multiplied by 100,000. Youth arrest rates for violent offenses from 2012 to 2021 are reported in the Supplemental Materials Table 1 for the two intervention communities and the remaining 76 non-intervention communities in Denver.

### Analysis Plan

We conducted descriptive analyses of youth arrests for violent offenses by analyzing trends in the two intervention communities compared to the combination of all remaining 76 Denver communities that were not part of the intervention. To proceed with more rigorous methods for evaluating the intervention effects on youth violence, we implemented two quasi-experimental approaches: (1) ITS and (2) DiD with synthetic controls. Both models use linear regression estimation techniques and natural logarithmic transformations of the youth arrest rates as the dependent variables. This is a common transformation of rate variables that tend to have highly skewed distributions. It improves the fit of the model and still yields a straightforward conceptual interpretation (Osgood, 2000). See Supplemental Materials Tables 2 and 3 for the data used in the ITS and DiD models, respectively.

First, to test for significant changes in youth violence after strategies were implemented, beyond what would be expected based on the pre-intervention trend, ITS was employed. It uses the trend before the intervention to control for non-randomness in the community and to show the immediate and sustained effects of the intervention over time. In each of the intervention communities, the ITS model tests whether there was a significant trend in arrest rates before and after the intervention was implemented, and if the pre- and post-trends are different. The natural logarithm of the arrest rates was the dependent variable in each model, and the predictor variables included the year of the study (0 = 2012, 1 = 2013,…, 9 = 2021); a dummy indicator of whether the intervention was in effect (0 = 2012–2016, 1 = 2017–2021); and a variable for the year after the intervention began (0 = 2012 thru 2016, 1 = 2017, 2 = 2018, 3 = 2019, 4 = 2020, 5 = 2021).

Second, we used DiD models with synthetic controls to rigorously test intervention effects over time. Because there were no closely comparable Denver communities to serve as controls for the intervention communities, the DiD models were estimated using synthetic controls for comparison. The Stata (StataCorp, 2024) “synth” procedure was used to derive a synthetic control for each intervention community, and it also produced the following synthetic control estimates for comparison to intervention Communities A and B. Faced with the challenge of evaluating intervention effects in the absence of appropriate control communities, the DiD with synthetic control design is the most robust quasi-experimental design (QED) available. Synthetic controls are widely used in evaluating place-based program or policy effects and involve creating a statistically derived single control community that is the best possible match to the intervention community based on its demographic characteristics as well as its youth arrest rates in the pre-intervention period (Abadie & Gardeazabal, 2003; Saunders et al., 2015).

Once the synthetic control is derived and its corresponding set of outcome values are calculated, the DiD regression model can be estimated by providing trend lines fitted to the data as well as tests for intervention effects. These effects are found if, during the intervention period (2017–2021), the estimated slope of the trend line for the intervention community is statistically more negative than for the synthetic control community, or if the intercept of the estimated trend line for the intervention period, i.e., the estimated natural logarithm of the arrest rate in 2017, is significantly lower for the intervention community than for the synthetic control community. After the synthetic control was constructed, the DiD model was estimated with the violence outcomes data for the intervention and synthetic control communities, and then tests were used to evaluate if the changes in the outcomes after the intervention were significantly different for each intervention community compared to its synthetic control.

## Results

### Implementation Process Evaluation

#### Readiness Capacity Assessment and Intervention Fidelity

Of the 137 evaluations received from Community Board, Key Leader and Workgroup meetings (approximately 90 meetings), data showed that coalition members felt the meetings were overall productive (M = 3.5 on a 1–4 scale), and they were satisfied with the quality of the meetings (M = 4.5 on 1–5 scale). Scores were similar across communities, with slightly higher scale means in Community B.

Next, each coalition participated in a community coalition survey that included measures of readiness to perform their functions in monitoring and supporting the strategy implementations in each community. The survey was based on an existing, well-known organizational readiness tool (Scaccia et al., 2015), which was adapted for community coalitions in the current study. Coalition surveys were conducted once a year in 2018, 2020, and 2021; the survey was modified in 2019 and was not administered during that year. Results from the surveys indicated high levels of readiness during each year of implementation, with capacity scores ranging from 73 to 86% across all years and both communities. Both coalitions indicated moderate levels of motivation (58-69%); moderate to high levels of opportunities for participation (62-98%); and high levels of cohesion (81-94%), support for a science-based approach (86-97%), and community transformation (73-100%). Scores on the diversity of stakeholders were mixed (57-78%), and varied from year to year, but were generally higher among the Community B coalition members.

Finally, a Milestones and Benchmarks (Quinby et al., 2008) assessment was completed for each phase of the CTC initiative to track fidelity to the CTC model. The assessment was completed by coalition board co-chairs/representatives from each intervention site, the YVPC-D Project Director, and the Community Site Representatives, typically four people per site. The average respondent ratings for completion of each phase 1–5 milestone benchmark ranged from 82 to 90% for Community A and 73–97% for Community B.

*Community prevention strategy reach.* Across the intervention communities, the following numbers are provided to estimate the community participant reach and number served for each prevention strategy: over 3,000 youth and adults for the PO1 media campaign across six events (Community A and B); about 7,000 youth and families were served by the Mini Grants, and at least 31 youth received direct recognition through two Positive Recognition Campaign events (Community A); over 1,000 youth and families participated in PATHS/Community SEL (Community B); and 2,109 youth and families were screened through the VIPRS tool (Community A and B). Overall, the collective strategies served at least 13,140 youth and families in Communities A and B.

### Descriptive Analyses of Youth Violence Outcomes

**Fig. 1:**
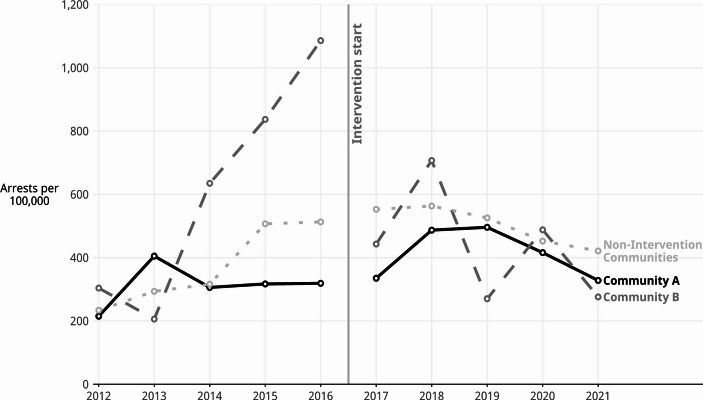
Annual youth arrests for violent offenses per 100,000 youth (aged 10–24) by intervention community in Denver, CO, 2012–2021. Data source: Denver Police Department arrest incident data

Figure 1 displays the annual youth arrest rates for violent crimes over the study period in the intervention and non-intervention area, i.e., the combination of the remaining 76 communities in Denver that were not part of the intervention. Visual inspection of the figure reveals that the most substantial differences were between the two intervention communities A and B. Both began (in 2012) and ended (in 2021) with roughly similar arrest rates. However, Community B experienced increasing arrest rates before the intervention and then decreasing rates after the intervention. In contrast, the arrest rates in Community A remained relatively low and, for the most part, did not exhibit any dramatic increasing or decreasing trends. Community A had substantially lower arrest rates for violent crimes in the last three of the five years of the pre-intervention period compared to Community B and the non-intervention area (City and County of Denver, n.d.). Moreover, from 2013 to 2016, Community A rates were stable and not increasing; however, the arrest rates for youth violent crimes in Community B increased dramatically from 206 per 100,000 youth in 2013 to 1086 per 100,000 youth by 2016. The arrest rate trends for the non-intervention areas fell between the rates of two intervention communities. Arrest rates for the remainder of Denver, i.e., the non-intervention area, increased more gradually than in Community B in the pre-intervention years. In the post-intervention years, the rates for the non-intervention area decreased gradually but remained consistently higher than in Community A.

In the first year of the intervention, the trend of increasing arrest rates in Community B reversed and the arrest rate fell from 1,086 per 100,000 youths in 2016 to 443 per 100,000 youths in 2017. In intervention Community A, and in the non-intervention communities, the arrest rates in 2017 remained similar to where they were before the intervention in 2016. In the years during the interventions, the arrest rates varied widely in Community B but, overall, followed a decreasing trend. The arrest rates during the intervention were u-shaped in Community A with a moderate increase from 335 per 100,000 in 2017 to 496 per 100,000 in 2019 and then back down to 328 per 100,000 in 2021. Throughout the intervention period, the arrest rates for Community A remained lower than in the non-intervention area.

As part of the descriptive analysis, we compared the mean arrest rates in the pre-intervention period to those in the post-intervention period. On average, arrest rates in Community A increased 33% from 312 to 412 arrests per 100,000 youth. The next highest average increase was in the combined non-intervention areas, which increased 35% from 373 to 503 arrests per 100,000. Of the three communities, Community B was the only one to decrease, and, on average, decreased 29% from 613 to 436 arrests per 100,000 youth.

**Fig. 2:**
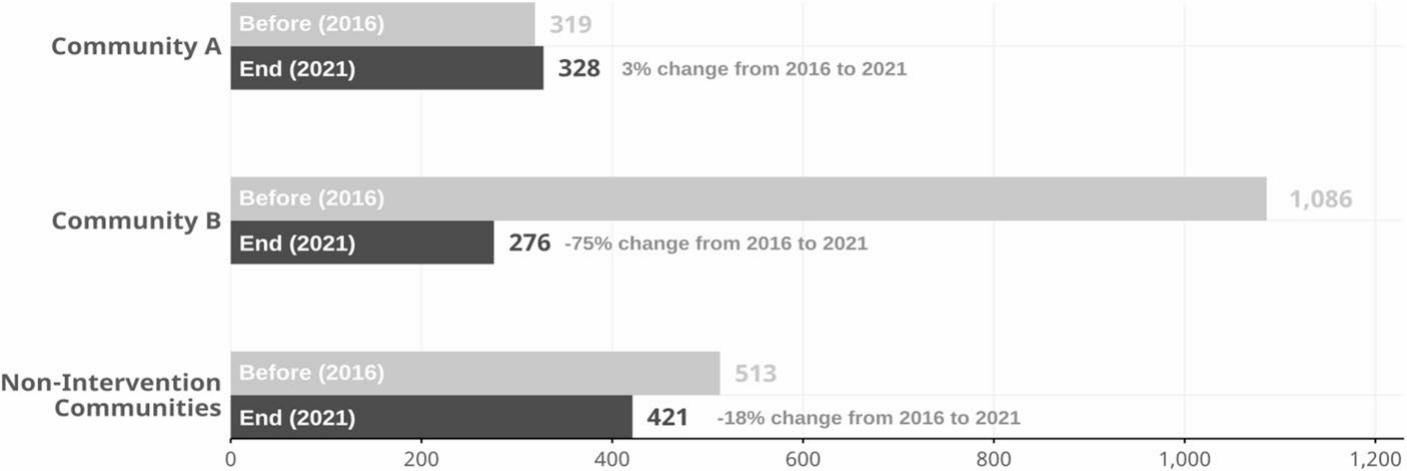
Youth arrests for violent offenses per 100,000 youths (aged 10–24) before intervention (2016) and at end of intervention (2021), City of Denver, CO. Data source: Denver Police Department arrest incident data

While the mean comparisons are valuable, they tend to disguise the trends that are visible in the data, and they may not fully characterize the differences between the pre-intervention and post-intervention periods. Another way to describe the intervention effects is to compare arrest rates in the year just before the intervention began (2016) with rates at the end of intervention period (2021). These are reported in Fig. 2, and from this analysis the arrest rates in Community A remained stable and relatively unaffected by the intervention, whereas Community B decreased 75% from 1,086 to 276 arrests per 100,000 youth. The combination of all non-intervention communities reveals the predominate trend in the City of Denver, which was an 18% decrease in arrest rates for violent offenses from 513 in 2016 to 421 per 100,000 youth in 2021.

### Interrupted time Series Analysis

**Fig. 3:**
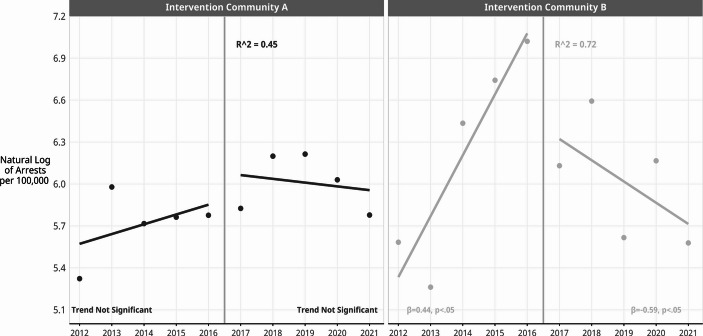
Within-community trend lines fitted to youth arrest rates for violent offenses before and after intervention, with ITS effects, Intervention Communities A and B, 2012 to 2021, City of Denver, CO. Data source: Denver Police Department arrest incident data

The ITS models included five years of data from the pre-intervention period (2012–2016) and five years of data (2017–2021) from the intervention period, and they were estimated separately for intervention Community A and for intervention Community B. The within-community results (see Fig. 3) show the effects estimated by the ITS models.

The ITS models fit trend lines to the pre-intervention data, and another trend line was fitted to the post-intervention data. The slope in the pre-intervention years in Community A was slightly increasing, but not significantly different from zero (β = 0.06, *p* =.49). Community A shows a slightly negative trend in arrests during the intervention (β=-0.07, *p* =.50), but it also was not significantly different from zero. In Community B, arrest rates increased significantly in the pre-intervention years (β = 0.44, *p* <.05), and then decreased significantly in the post-intervention years (β=-0.59, *p* <.05).

### Evaluation of Intervention Effects: Difference in Differences (DiD) with Synthetic Controls

**Fig. 4:**
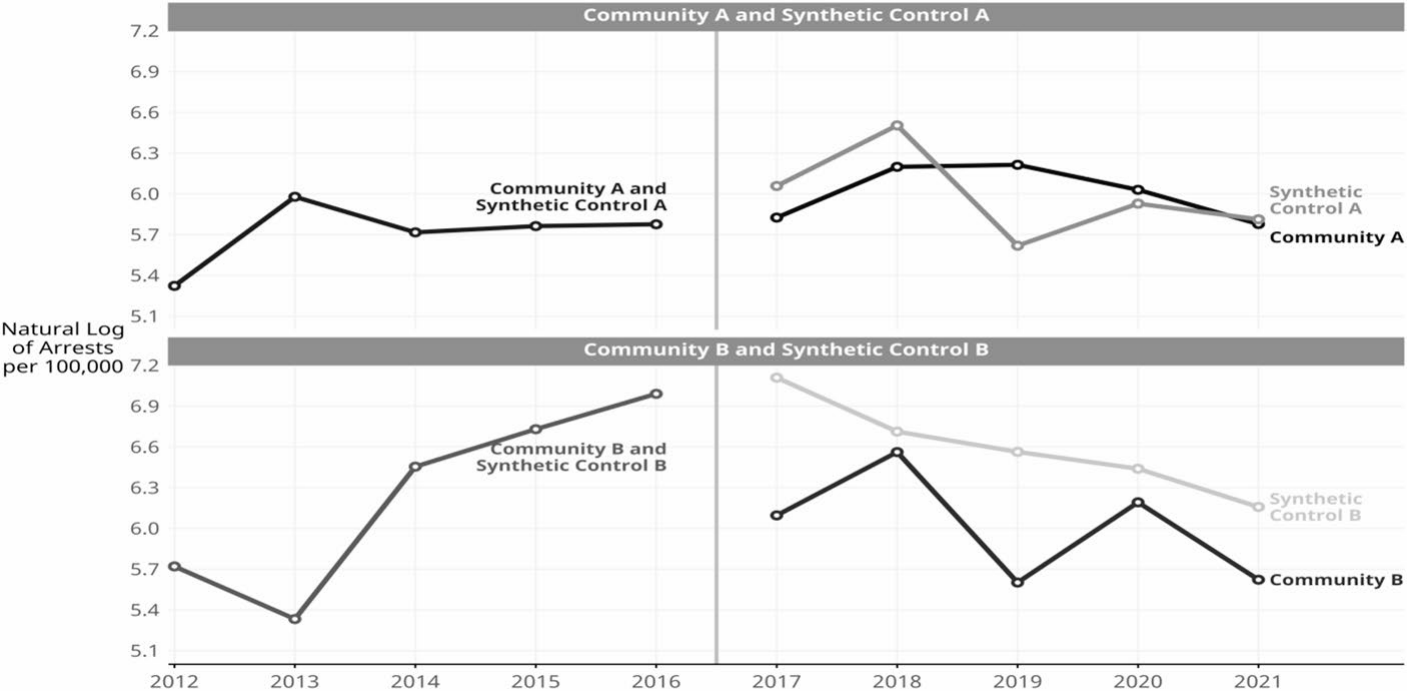
Youth arrest rates for violent offenses for intervention communities and synthetic control communities, Denver, Colorado, 2012–2021. Data source: Denver Police Department arrest incident data

Synthetic controls were statistically derived from the remaining 76 non-intervention communities in the City of Denver using community-level known predictors of arrests for violent offenses and these were aggregated from the U.S. Census tract-level 5-year ACS estimates for the years 2012–2016. The chosen predictors were: (1) proportion White, (2) proportion non-Hispanic Black, (3) proportion Hispanic, (4) proportion in poverty, and (5) proportion unemployed. In the end, only proportion in poverty and proportion unemployed were statistically important in deriving the synthetic control communities. Also, two Denver communities had incomplete predictor data and they were eliminated from the synthetic control analysis at the outset. This reduced the number of donor communities to 74. Figure 4 shows the values that resulted for the synthetic controls and how they compare to the intervention communities.

**Fig. 5:**
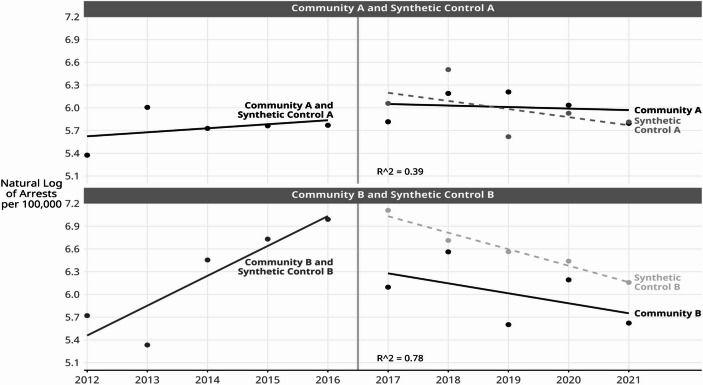
Fitted youth arrest rates for violent offenses trends lines for intervention and synthetic control communities: Estimated with DiD model, Denver, Colorado, 2012–2021. Data source: Denver Police Department arrest incident data

#### DiD Intervention Results for Community A

Figure 5 shows the fitted trend lines, estimated with the DiD model, before and after the intervention began, and the DiD estimates and test statistics are reported in Table 2. Since the arrest rate values are virtually identical for the intervention and for its synthetic control in the years before the intervention, the model is most useful for testing for differences in the characteristics of the trend lines fitted to the data during the intervention period. The DiD results for Community A are displayed in the top panel of Fig. 5. The trend lines representing the arrest rates in the intervention period (2017–2021) are decreasing slightly for both communities (β=-0.08 for Community A and β=-0.11 for its synthetic control). However, the slopes of the trend lines are not significantly different from zero, which implies that statistically there is no trend.

**Table 2:** Tests for differences in youth arrests for violent offenses during intervention years (2017–2021): intervention communities versus synthetic control

	**Community A versus Synthetic Control A**					
	DiD Estimated Difference in Intercepts			DiD Estimated Differences in Slopes		
Natural Logarithm of Arrests per 100,000	Community A	Synthetic Control	Joint F-test (2,12) for difference in intercepts	Community A	Synthetic Control	Joint F-test (2,12) for difference in intercepts
	6.31	6.07	1.10, ns	-0.08	-0.11	0.28, ns
Arrests per 100,000	549	432		-1.08	-1.12	
	**Community B versus Synthetic Control B**					
Natural Logarithm of Arrests per 100,000	DiD Estimated Difference in Intercepts			DiD Estimated Differences in Slopes		
	Community B	Synthetic Control	Joint F-test (2,12) for difference in intercepts	Community B	Synthetic Control	Joint F-test (2,12) for difference in intercepts
	6.41	7.25	10.39, *p* <.002	-0.13	-0.22	1.73, ns
Arrests per 100,000	607	1407		-1.14	-1.25	

Note: DiD = Differences in Differences. ns = Not statistically significant. Data source: Denver Police Department arrest incident data

In addition, the post-intervention trend lines are not significantly different from each other, and the intercepts of the trend lines after intervention began are not significantly different either. These tests were conducted with joint F-tests and the results are reported in Table 2, both in natural logarithm units as well as number of arrests per 100,000. The 2017 estimated number of arrests per 100,000 was 549 for Community A compared with 432 for its synthetic control, and the estimated annual decrease from 2017 to 2021 was 1.08 arrests per 100,000 in Community A compared with 1.12 arrests per 100,000 in the synthetic control. The goodness of fit for this DiD model is R^2^ = 0.39 and this suggests that this model is not a particularly good representation of the data, most likely because there was no post-intervention linear trend in Community A or in its synthetic control.

#### DiD Intervention Results for Community B

The lower panel of Fig. 5 shows the fitted regression lines before and after intervention for Community B and for its synthetic control. As in Community A, the pre-intervention data points and fitted trend lines are virtually identical for Community B and its synthetic control. However, the DiD estimated intercepts for the intervention trend lines, i.e., one year after the intervention began, were 6.41 for Community B and 7.25 for its synthetic control. Translating from natural logarithms back to the original units of arrests per 100,000, in 2017 the estimated arrest rate for Community B was 607 arrests per 100,000 versus 1,407 per 100,000 for its synthetic control. A joint F-test (2, 12) confirmed that the intercepts of the two regression lines during the intervention period were significantly different (F = 10.39, *p* <.002). These results are reported in Table 2.

Each of the slopes of the estimated trend lines declined after the intervention (β=-0.13 for Community B and β=-0.22 for its synthetic control) but the slopes were not significantly different from each other (F(2,12) = 1.73, ns). These slopes translate into an annual estimated decrease of 1.14 arrests per 100,000 in Community B and 1.25 in its synthetic control. The R^2^ = 0.78 indicates that this model is a good representation of the data.

## Discussion

Overall, the descriptive results are consistent with the statistical tests from the ITS and the DiD models showing that arrest outcomes for Community A remained fairly low and stable before and after the intervention. This finding is especially apparent relative to intervention Community B and relative to the combined areas of Denver that did not receive the intervention. The ITS tests confirm that the slopes of the arrest trends in Community A were not significantly different from zero before and after the intervention, and the DiD results confirm that the arrest trend for Community A did not differ significantly from its synthetic control community, which can be interpreted as the expected trend for Community A without any intervention. In contrast, the descriptive results show that there was an increase in arrests in the non-intervention area of Denver during the first five years of the study, and there was also a prevailing decline in arrest rates in the last five years of the study, coincidentally during the intervention. However, arrests in Community B were increasing more notably before the intervention and decreased more sharply after the intervention than the combined non-intervention areas of Denver or intervention Community A.

The ITS trends fitted to the arrest rates in Community B confirm the visual impression that the arrests trends were substantially different in the pre-intervention years compared to the intervention years, suggesting a positive intervention effect in Community B. The DiD test results further substantiate the intervention effect in Community B by confirming that the intercept of the fitted trend line for Community B was significantly lower than for the synthetic control community, indicating lower levels of youth violence beginning in 2017, one year after the intervention began, and continuing throughout the intervention period. An important additional DiD result is that the trend lines for intervention Community B and its synthetic control community are both decreasing during the intervention years and the slopes of the two regression lines are not significantly different. This finding underscores that the intervention effect identified in this study is not the decreasing arrest trend that is found in both the intervention Community B and its synthetic control. However, the decreasing arrest trends, over a longer period of time (2017–2021), may be partially due to external non-intervention effects experienced by all high-burden, high-crime communities throughout the city.

This study contributes to the limited evidence base on community-level violence prevention strategies by testing the within and between community effects of the CTC prevention system and community-selected strategies effects on reducing youth violence in two Denver communities experiencing high-burden. Unlike most CTC research, it also includes process evaluation data to describe coalition functioning and implementation reach. By using the DiD with synthetic control design, this research rigorously tests the intervention effects using a robust QED. Additionally, by reporting youth and community violence trends during the COVID-19 pandemic, it adds an important context to the youth and community violence and prevention literature base.

The results show there were no intervention effects in Community A. However, it is important to keep in mind that Community A was a community experiencing high-burden with arrest rates for youth that were relatively low before the intervention began. This may be attributed to its participation as an intervention community (that targeted a similar population and aimed to reduce the same outcome) in a recent cycle of funding just prior to the beginning of this study. The stable arrest rates observed in Community A could also be a marker of community resilience during the COVID-19 pandemic (Alderden & Perez, 2021).

Community B, on the other hand, had youth arrests for violent offenses that were increasing at a substantial rate in the five years before the intervention, with lower youth arrest rates observed after the intervention began. After the initial decline, arrest rates in Community B continued to decline and remained significantly lower, on average, than the synthetic control community throughout the intervention period. The intervention effects in Community B suggest that the CTC prevention system combined with community-level prevention strategies that focus on the outer socio-ecological levels (i.e., community, societal) may have the potential to impact community rates of youth violence measured as reductions in youth arrests for violent offenses.

The positive intervention effects observed in Community B are consistent with results from other CTC evaluations. For example, this study provides evidence that CTC can be effectively implemented and reduce violence outcomes in urban settings (Brady et al., 2018). This project adds to the CTC evidence base of reduced community-level youth substance use, antisocial behavior, and internalizing symptoms (Chilenski et al., 2019; Feinberg et al., 2010; Hawkins et al., 2014; Toumbourou et al., 2019) and youth handgun carrying (Rowhani-Rahbar et al., 2023) by showing similar positive intervention effects on lower youth violence arrest rates.

### CTC Implementation in Intervention Communities

There were some shared challenges in how CTC was implemented in Community A and Community B during the project period. First, both urban communities were comprised primarily of residents from racial and ethnic minority groups. The CTC videos that were intended to be used to train coalition members in the CTC process were not well received by coalition members in either community, as members did not feel the videos contained enough people from racial and ethnic minority groups and did not deliver content in ways that were culturally relevant. Community A abandoned the use of CTC videos after less than a year, and Community B did not use them at all. Second, the amount of time recommended by CTC to effectively train coalition members was unrealistic for many members. CTC recommends a two-day training orientation for new coalition members, and at least a day-long workshop for members as they go through each phase of CTC. Coalition members could not fully make this time commitment, and so the CTC facilitators frequently found themselves modifying the CTC model to distill key CTC concepts and share them with coalition members in abbreviated ways. Third, the COVID-19 pandemic also impacted implementation of CTC in both communities. For example, the pandemic impacted communities needing to adjust their programming and services due to access issues, including virtual and hybrid formats; and youth engagement and attendance in both virtual and live events.

Several characteristics of the communities may help explain the differing intervention effects in Communities A and B. Community A had many contextual and practical implementation challenges that were different from Community B, which differentially affected the CTC process and fidelity of implementation. For example, Community A had evolved significantly between 2011 and 2016, as it added almost 10,000 more residents, underwent a large educational reform effort, incorporated a new transportation infrastructure, and began experiencing the same impacts of gentrification and rising housing process as the rest of the City and County of Denver. Increased competition resulted in duplicated efforts to convene and organize residents, which created tension between the CTC coalition and other organizations in the community. Based on qualitative data, Community A experienced more tensions and personality conflicts between key staff than Community B, which resulted in diminished morale and enthusiasm of some coalition members. Further, there were residential size differences between communities, and Community A may have had more limited intervention reach due to its larger size. Although there was mobilization in both communities, Community B was characterized as considerably smaller in population size, allowing more personal connections and better cohesion among community members. Early-on organization in Community B was activated by strong coordinators and leadership, and there was more law enforcement presence bolstered by partnerships between law enforcement and community organizations.

There were also several differences in how CTC was implemented between the two communities, primarily due to the unique environmental contexts of each area. Community A had been implementing CTC since 2011 and the coalition had been running for almost five years before the start of the project in 2016, while Community B operated as a comparison community during that time and participated in data collection but did not implement CTC or any of the funded interventions. Therefore, CTC was new to Community B in this project. Community B had to build their coalition during this project period. They had begun to organize after some significant violent events that took place in the community between 2008 and 2014. Consequently, the energy and enthusiasm of Community B to implement CTC was stronger than in Community A.

As previously mentioned, Community A is almost four times the size of Community B, yet the size of both coalitions was similar, which allowed Community B’s coalition and programming to potentially have more reach and impact on their community than Community (A). The larger size of Community A also made community-wide strategy implementation more difficult. Proportionately, fewer youth were served by the strategies in Community A compared to Community B, particularly as the schools in Community A were not implementing a universal-level program as Community B did. While both communities went through some degree of educational turnaround and reform, the amount of change was much more pronounced in Community A than Community (B). Community A struggled to maintain relationships with key school personnel as leadership and staff at most schools changed constantly from year to year. Some of this turnover also occurred in Community B, but not to the same extent, and the coalition was more successful in engaging key school personnel in coalition meetings and to support their implemented strategies.

### Limitations and Future Directions

Although this study has several strengths, it also has limitations. First, the intervention effect on fewer youth arrests for violent offenses found in Community B (and lack of intervention effect in Community A) could be influenced by other external and internal non-intervention factors, a common issue in QEDs. The intervention took place during the COVID-19 pandemic, a period of increased gentrification and civil unrest in urban communities, which may have contributed to lower law enforcement presence and decreased arrests, as well as violence being displaced to other parts of the city. Additionally, in the years before the CTC intervention, youth arrests increased steadily in both the intervention and synthetic control community, so another explanation could be that due to elevated levels of arrests and subsequent incarcerations, fewer high-risk youth were left in the communities and therefore youth violent offenses decreased. Likewise, other unmeasured violence prevention efforts may have contributed to the reductions of violence in Community B. One final explanation is that during the intervention period, neighborhood social processes that may be associated with reductions in youth violence improved in Community B and to some extent in other disadvantaged neighborhoods throughout the city as well, which could partially dilute some of the observed intervention effects. Associations between community-level violence prevention strategies and neighborhood social processes (e.g., ethnic-racial identity, institutional quality, collective efficacy) are currently being explored in a subsequent paper to further help understand intervention effects. Overall, despite these possible alternative explanations for study findings, we believe the intervention effect of lower arrest rates for Community B is meaningful and remains the most plausible explanation given the rigorous design and analysis methods we implemented (DiD models with synthetic controls) to exclude potential confounders.

Second, Community A was in its second phase of implementing interventions due to a prior CTC grant (Kingston et al., 2016a, b; Kingston, Mihalic et al., 2016); analyses do not account for prior CTC implementation and potential effects on youth arrest rates due to non-compatibility of pre-2010 data with the current study’s post-2010 data. Third, the length of time for full implementation of the intervention was shorter than planned (e.g., for the PO1 campaign, which had difficulties getting launched and was impacted in multiple ways by the COVID-19 pandemic). Thus, some prevention strategies may not have been implemented at the ideal length of time for adequate dosage and impact. Other CTC evaluations indicate that the effects of community-level interventions on behavioral outcomes can take 4–10 years to be observed (e.g., Hawkins et al., 1992; Oesterle et al., 2015). Therefore, it is possible that positive effects of the prevention strategies may still emerge over time. Further, there were residential size differences between communities, and Community A may have had more limited intervention reach due to its larger size. Fourth, this study does not tease out which components of the prevention infrastructure account for impact. Fifth, this study used only official arrest data for its primary violence outcome. Arrests are tied to police enforcement, investigations, and follow through, which could bias the data; police-reported violent offenses would be preferred as an outcome, but these data were not available with the youth ages of the offender. Therefore, this study could not isolate youth violence offenses. Selected CTC strategies in Community A and B were not identical which may have also contributed to significant intervention effects in one community but not the other. Lastly, the study could be further strengthened by examining other measures of youth violence such as emergency room visits for injury, school fighting, and self-reported data. However, funding resource limitations and other practical reasons prevented this additional data collection.

We have identified several recommendations for further research related to this study. First, more research is needed on CTC implementation in urban communities of people from racial and ethnic minority groups to better understand how to address their unique needs, including identifying and using culturally relevant qualitative and quantitative research methods. Second, additional research is needed to evaluate community-level prevention strategies to understand which strategies are most effective in preventing youth violence across different communities. Third, future research should consider expanding the measurement of outcome variables beyond official arrest data and measure other relevant neighborhood and contextual variables to better understand what processes impacted the study results. For example, community gentrification, protest, and civil unrest may be factors that influenced the study outcomes, as they impacted many communities in the United States during this time (D’Inverno & Bartholow, 2021; Mendoza-Graf et al., 2023; Rosenfeld & Lopez, 2020, 2022). Fourth, while this project contributed to understanding the impact of prevention strategies focused on the outer socio-ecological levels, combining interventions targeting the individual, family, and community levels is likely to have the greatest impact on youth violence. Future research is needed to develop and test the impact of the full multi-tiered model of a comprehensive public health approach on youth violence. Finally, the monumental work of building sustained violence prevention infrastructures in urban communities of people from racial and ethnic minority groups experiencing high burden is challenging. For example, local grassroot community-based organizations bear the burden of addressing these pressing social problems and have demonstrated strong effects on reducing crime and violence, yet they require resources and support to continue this work (Buggs, 2022; Buggs et al., 2022; Cahill & Hayeslip, 2010; Pugliese et al., 2022; Sharkey et al., 2017). Long-term sources of support can help ensure research for this work is sustained.

## Conclusions

This study suggests that community-level approaches focused on outer levels of the social ecology have the potential to prevent youth violence. Further, the study provides evidence that the CTC prevention system can be successfully implemented in an urban setting and can contribute to lower rates of community youth violence. The intervention effect in Community B was observed early in the intervention period, suggesting that the mobilization period was impacting the intervention community with respect to youth arrest rates. The lack of intervention effects in Community A were possibly due to several practical and contextual influences (e.g., it may have been more difficult for CTC to be implemented and have a strong impact in the larger, more populated geographic region, implementing programs that are community-developed and not necessarily dissemination-ready may take more time). Additionally, challenges with coalition functioning may have impacted program implementation and outcomes in Community A. Given that the negative and costly consequences of violence on young people’s development and community functioning are magnified in urban settings experiencing high-burden, identifying solutions that reduce community youth violence can significantly improve public health.

## Electronic Supplementary Material

Below is the link to the electronic supplementary material.

Supplementary Material 1
