# Supplementary material for: Effects of the Communities that Care (CTC) Prevention System on Youth Violence Outcomes in Two Violence-Impacted Denver Communities
Source: Am J Crim Justice. 2025 Jul 12;51(1):281–308. doi: 10.1007/s12103-025-09811-0 (PMC12950059; doi:10.1007/s12103-025-09811-0)
Supplement: Supplementary file 1 — Supplementary Material 1 [file 12103_2025_9811_MOESM1_ESM.docx]

Supplementary Materials

| Supplemental Table 1. Youth Arrest Rates for Violent Offenses for Denver Communities, 2012-2021 | | | | | | | | | | |
| --- | --- | --- | --- | --- | --- | --- | --- | --- | --- | --- |
|  | **2012** | **2013** | **2014** | **2015** | **2016** | **2017** | **2018** | **2019** | **2020** | **2021** |
| Community A | 215 | 405 | 306 | 317 | 319 | 335 | 487 | 496 | 416 | 328 |
| Community B | 304 | 206 | 635 | 837 | 1086 | 443 | 707 | 270 | 488 | 276 |
| Non-intervention Communities Combined | 234 | 294 | 316 | 507 | 513 | 553 | 563 | 526 | 453 | 421 |
| 76 Non-intervention Communities |  |  |  |  |  |  |  |  |  |  |
| 1 | 111 | 223 | 674 | 453 | 285 | 461 | 175 | 589 | 179 | 181 |
| 2 | 263 | 0 | 172 | 153 | 568 | 680 | 269 | 817 | 142 | 153 |
| 3 | 109 | 386 | 1029 | 637 | 807 | 1068 | 272 | 349 | 487 | 161 |
| 4 | 190 | 465 | 441 | 755 | 649 | 334 | 177 | 778 | 223 | 365 |
| 5 | 80 | 304 | 146 | 212 | 138 | 408 | 337 | 134 | 135 | 342 |
| 6 | 95 | 51 | 327 | 236 | 639 | 139 | 764 | 169 | 189 | 213 |
| 7 | 0 | 0 | 305 | 283 | 0 | 0 | 0 | 503 | 507 | 0 |
| 8 | 299 | 338 | 373 | 133 | 552 | 274 | 524 | 363 | 434 | 96 |
| 9 | 81 | 172 | 92 | 645 | 758 | 838 | 1078 | 777 | 924 | 1268 |
| 10 | 693 | 2413 | 1764 | 3718 | 3128 | 3021 | 4205 | 2649 | 2053 | 2583 |
| 11 | 557 | 0 | 0 | 154 | 0 | 317 | 168 | 0 | 859 | 535 |
| 12 | 200 | 111 | 0 | 931 | 287 | 153 | 160 | 656 | 0 | 485 |
| 13 | 0 | 202 | 0 | 183 | 359 | 358 | 545 | 0 | 198 | 0 |
| 14 | 610 | 0 | 692 | 738 | 2365 | 421 | 450 | 1442 | 1027 | 0 |
| 15 | 0 | 1041 | 0 | 551 | 191 | 602 | 1279 | 2289 | 1247 | 1384 |
| 16 | 20640 | 20810 | 8609 | 32840 | 18677 | 31706 | 28413 | 19730 | 8833 | 15135 |
| 17 | 0 | 185 | 282 | 766 | 488 | 200 | 307 | 105 | 108 | 336 |
| 18 | 233 | 88 | 296 | 437 | 1299 | 372 | 1260 | 371 | 586 | 216 |
| 19 | 331 | 659 | 679 | 771 | 648 | 610 | 540 | 680 | 98 | 102 |
| 20 | 0 | 0 | 137 | 74 | 157 | 409 | 251 | 83 | 406 | 155 |
| 21 | 0 | 0 | 0 | 395 | 0 | 175 | 0 | 0 | 184 | 0 |
| 22 | 0 | 0 | 0 | 0 | 0 | 0 | 0 | 0 | 0 | 0 |
| 23 | 381 | 872 | 0 | 498 | 976 | 1356 | 1601 | 2752 | 2908 | 1222 |
| 24 | 250 | 122 | 359 | 517 | 600 | 489 | 758 | 396 | 327 | 760 |
| 25 | 0 | 58 | 177 | 298 | 419 | 297 | 293 | 400 | 553 | 531 |
| 26 | 253 | 330 | 578 | 469 | 1034 | 1176 | 884 | 563 | 814 | 666 |
| 27 | 63 | 64 | 65 | 0 | 137 | 282 | 438 | 304 | 159 | 167 |
| 28 | 153 | 152 | 273 | 227 | 104 | 150 | 311 | 376 | 270 | 174 |
| 29 | 665 | 532 | 225 | 394 | 711 | 409 | 306 | 217 | 348 | 472 |
| 30 | 187 | 305 | 219 | 699 | 487 | 750 | 126 | 0 | 360 | 799 |
| 31 | 88 | 0 | 146 | 351 | 569 | 359 | 455 | 239 | 92 | 71 |
| 32 | 0 | 182 | 247 | 38 | 436 | 352 | 416 | 556 | 354 | 257 |
| 33 | 0 | 57 | 123 | 0 | 313 | 120 | 277 | 0 | 132 | 77 |
| 34 | 148 | 77 | 118 | 81 | 370 | 291 | 376 | 458 | 330 | 122 |
| 35 | 118 | 0 | 104 | 302 | 197 | 393 | 198 | 203 | 106 | 280 |
| 36 | 85 | 616 | 271 | 553 | 467 | 658 | 282 | 467 | 185 | 0 |
| 37 | 186 | 579 | 0 | 200 | 1592 | 196 | 190 | 182 | 0 | 0 |
| 38 | 0 | 0 | 0 | 0 | 0 | 0 | 0 | 0 | 0 | 0 |
| 39 | 0 | 231 | 248 | 797 | 283 | 598 | 939 | 974 | 333 | 1010 |
| 40 | 0 | 0 | 111 | 0 | 209 | 307 | 504 | 600 | 200 | 402 |
| 41 | 500 | 406 | 80 | 1028 | 1423 | 1435 | 1621 | 1331 | 1553 | 1175 |
| 42 | 160 | 105 | 258 | 202 | 198 | 241 | 0 | 0 | 266 | 259 |
| 43 | 314 | 198 | 205 | 244 | 490 | 242 | 436 | 288 | 302 | 141 |
| 44 | 0 | 170 | 0 | 132 | 211 | 296 | 308 | 79 | 160 | 80 |
| 45 | 0 | 221 | 0 | 141 | 303 | 155 | 0 | 142 | 382 | 223 |
| 46 | 185 | 209 | 1287 | 1295 | 705 | 1349 | 1086 | 1557 | 1212 | 1719 |
| 47 | 544 | 0 | 99 | 1033 | 506 | 280 | 329 | 70 | 413 | 0 |
| 48 | 752 | 1012 | 205 | 366 | 176 | 360 | 988 | 237 | 1330 | 1383 |
| 49 | 176 | 188 | 400 | 426 | 454 | 966 | 0 | 272 | 288 | 609 |
| 50 | 0 | 0 | 0 | 83 | 0 | 77 | 73 | 0 | 64 | 59 |
| 51 | 0 | 0 | 0 | 0 | 0 | 594 | 0 | 0 | 0 | 546 |
| 52 | 322 | 366 | 248 | 429 | 190 | 192 | 361 | 218 | 349 | 795 |
| 53 | 198 | 826 | 0 | 233 | 755 | 553 | 0 | 0 | 0 | 0 |
| 54 | 210 | 0 | 0 | 116 | 348 | 455 | 0 | 419 | 295 | 183 |
| 55 | 215 | 352 | 208 | 341 | 268 | 263 | 193 | 316 | 124 | 182 |
| 56 | 412 | 131 | 126 | 244 | 0 | 0 | 343 | 0 | 0 | 0 |
| 57 | 0 | 0 | 90 | 173 | 86 | 435 | 182 | 0 | 0 | 129 |
| 58 | 96 | 846 | 499 | 826 | 551 | 569 | 418 | 645 | 366 | 303 |
| 59 | 1458 | 2244 | 1776 | 3838 | 1790 | 3544 | 2236 | 1450 | 1634 | 672 |
| 60 | 437 | 505 | 514 | 591 | 541 | 561 | 513 | 1004 | 657 | 88 |
| 61 | 1487 | 844 | 2851 | 8216 | 6785 | 4208 | 3229 | 2027 | 2884 | 2560 |
| 62 | 0 | 26 | 78 | 132 | 27 | 0 | 55 | 84 | 57 | 58 |
| 63 | 0 | 0 | 394 | 151 | 520 | 0 | 216 | 695 | 240 | 475 |
| 64 | 0 | 159 | 0 | 147 | 0 | 48 | 0 | 48 | 49 | 50 |
| 65 | 355 | 486 | 246 | 368 | 363 | 469 | 113 | 534 | 302 | 563 |
| 66 | 238 | 256 | 326 | 853 | 411 | 238 | 417 | 409 | 507 | 161 |
| 67 | 87 | 85 | 42 | 43 | 43 | 89 | 139 | 342 | 52 | 344 |
| 68 | 395 | 146 | 782 | 322 | 1273 | 452 | 550 | 245 | 214 | 185 |
| 69 | 201 | 216 | 0 | 0 | 0 | 196 | 176 | 155 | 0 | 0 |
| 70 | 192 | 363 | 172 | 219 | 210 | 505 | 438 | 47 | 137 | 221 |
| 71 | 1468 | 0 | 0 | 0 | 0 | 0 | 0 | 0 | 0 | 151 |
| 72 | 122 | 233 | 453 | 225 | 683 | 765 | 933 | 1417 | 454 | 356 |
| 73 | 1067 | 139 | 422 | 279 | 135 | 515 | 240 | 331 | 101 | 91 |
| 74 | 186 | 328 | 520 | 573 | 435 | 441 | 523 | 457 | 337 | 397 |
| 75 | 119 | 1198 | 1233 | 1395 | 2875 | 1921 | 1376 | 1256 | 1279 | 754 |
| 76 | 0 | 118 | 61 | 369 | 123 | 122 | 120 | 349 | 56 | 106 |

Supplemental Table 2. Data Used in Interrupted Time Series (ITS) Models of the Intervention Effects on Youth Arrest Rates

|  | **Dependent Variables in ITS Models: Natural Logarithm of Arrests per 100,000** | | **Independent Variables in ITS Models** | | |
| --- | --- | --- | --- | --- | --- |
| Year | Community A | Community B | Year of Study | Intervention in Effect (0=no, 1=yes) | Year After Intervention Began |
| 2012 | 5.37 | 5.72 | 0 | 0 | 0 |
| 2013 | 6.00 | 5.33 | 1 | 0 | 0 |
| 2014 | 5.73 | 6.45 | 2 | 0 | 0 |
| 2015 | 5.76 | 6.73 | 3 | 0 | 0 |
| 2016 | 5.77 | 6.99 | 4 | 0 | 0 |
| 2017 | 5.81 | 6.09 | 5 | 1 | 1 |
| 2018 | 6.19 | 6.56 | 6 | 1 | 2 |
| 2019 | 6.21 | 5.60 | 7 | 1 | 3 |
| 2020 | 6.03 | 6.19 | 8 | 1 | 4 |
| 2021 | 5.79 | 5.62 | 9 | 1 | 5 |

Supplemental Table 3. Data Used in Difference in Differences (DiD) Models of the Intervention Effects on Youth Arrest Rates

|  | **Dependent Variables in DiD Models: Natural Logarithm of (Arrests per 100,000 + 1)** | | **Independent Variables in DiD Models** | | | | | | |
| --- | --- | --- | --- | --- | --- | --- | --- | --- | --- |
| Year | Community A | Community B | Year Relative to Beginning of Intervention | Year Relative to Beginning of Intervention for Intervention Community | Year after Intervention Began | Year after Intervention Began for Intervention Community | Intervention Community (1=yes, 0=synthetic control) | Intervention Year (1=yes, 0=no) | Intervention Year for Intervention Community (1=yes, 0=no) |
| 2012 | 5.38 | 5.72 | -4 | -4 | 0 | 0 | 1 | 0 | 0 |
| 2013 | 6.01 | 5.33 | -3 | -3 | 0 | 0 | 1 | 0 | 0 |
| 2014 | 5.73 | 6.45 | -2 | -2 | 0 | 0 | 1 | 0 | 0 |
| 2015 | 5.76 | 6.73 | -1 | -1 | 0 | 0 | 1 | 0 | 0 |
| 2016 | 5.77 | 6.99 | 0 | 0 | 0 | 0 | 1 | 0 | 0 |
| 2017 | 5.82 | 6.10 | 1 | 1 | 1 | 1 | 1 | 1 | 1 |
| 2018 | 6.19 | 6.56 | 2 | 2 | 2 | 2 | 1 | 1 | 1 |
| 2019 | 6.21 | 5.60 | 3 | 3 | 3 | 3 | 1 | 1 | 1 |
| 2020 | 6.03 | 6.19 | 4 | 4 | 4 | 4 | 1 | 1 | 1 |
| 2021 | 5.80 | 5.62 | 5 | 5 | 5 | 5 | 1 | 1 | 1 |
| Year | Synthetic Control for Community A | Synthetic Control for Community B | Year Relative to Beginning of Intervention | Year Relative to Beginning of Intervention for Intervention Community | Year after Intervention Year | Year after Intervention Year for Intervention Community | Intervention Community (1=yes, 0=synthetic control) | Intervention Year (1=yes, 0=no) | Intervention Year for Intervention Community (1=yes, 0=no) |
| 2012 | 5.39 | 5.73 | -4 | 0 | 0 | 0 | 0 | 0 | 0 |
| 2013 | 6.02 | 5.35 | -3 | 0 | 0 | 0 | 0 | 0 | 0 |
| 2014 | 5.75 | 6.46 | -2 | 0 | 0 | 0 | 0 | 0 | 0 |
| 2015 | 5.78 | 6.73 | -1 | 0 | 0 | 0 | 0 | 0 | 0 |
| 2016 | 5.78 | 6.99 | 0 | 0 | 0 | 0 | 0 | 0 | 0 |
| 2017 | 6.06 | 7.11 | 1 | 0 | 1 | 0 | 0 | 1 | 0 |
| 2018 | 6.50 | 6.71 | 2 | 0 | 2 | 0 | 0 | 1 | 0 |
| 2019 | 5.62 | 6.56 | 3 | 0 | 3 | 0 | 0 | 1 | 0 |
| 2020 | 5.93 | 6.44 | 4 | 0 | 4 | 0 | 0 | 1 | 0 |
| 2021 | 5.81 | 6.16 | 5 | 0 | 5 | 0 | 0 | 1 | 0 |
